# Children’s Processing and Comprehension of Complex Sentences Containing Temporal Connectives: The Influence of Memory on the Time Course of Accurate Responses

**DOI:** 10.1037/dev0000201

**Published:** 2016-10

**Authors:** Liam P. Blything, Kate Cain

**Affiliations:** 1 Department of Psychology, Lancaster University

**Keywords:** temporal connectives, incremental processing, memory, language acquisition, response times

## Abstract

In a touch-screen paradigm, we recorded 3- to 7-year-olds’ (*N* = 108) accuracy and response times (RTs) to assess their comprehension of 2-clause sentences containing *before* and *after*. Children were influenced by order: performance was most accurate when the presentation order of the 2 clauses matched the chronological order of events: “*She drank the juice, before she walked in the park*” (chronological order) versus “*Before she walked in the park, she drank the juice*” (reverse order). Differences in RTs for correct responses varied by sentence type: accurate responses were made more speedily for sentences that afforded an incremental processing of meaning. An independent measure of memory predicted this pattern of performance. We discuss these findings in relation to children’s knowledge of connective meaning and the processing requirements of sentences containing temporal connectives.

Successful comprehenders form a coherent mental representation of the events described in spoken or written text (Johnson-Laird, 1983; Van Dijk & Kintsch, 1983; Zwaan & Radvansky, 1998). The construction of a coherent mental representation is guided by the presence and understanding of connectives, which aid the integration of clauses by signaling how events link together (Bestgen & Costermans, 1997; Cozijn, Noordman, & Vonk, 2011). In this article, we focus on children’s processing of sentences containing the temporal connectives before and after, which encode the relation between events on a temporal dimension (Cain & Nash, 2011; Gennari, 2004). Whereas *before* and *after* appear regularly in speech from as young as 3 years of age (Diessel, 2004), 12-year-olds demonstrate difficulties in comprehending these connectives in specific sentence structures (Pyykkönen & Järvikivi, 2012). In the current study, we investigate the influence of memory and language on 3- to 7-year-old’s comprehension of complex sentences containing temporal connectives by investigating the influence of these skills on the accuracy and speed of responses using a touch-screen comprehension task.

Our mental representation of event order corresponds to the chronological order in which the events occur in real world situations: the first occurring event is followed by the second, and so forth (Coll-Florit & Gennari, 2011; Givón, 1991; Zwaan & Radvansky, 1998). However, the order in which events are described does not necessarily map onto actual order. Temporal connectives allow us to describe the events in both a chronological order, such as “*She played in the park, before she drank the juice*” and in a reverse order “*Before she drank the juice, she played in the park*.” Therefore, reverse order sentences violate the default expectation that newly encountered information follows the most recent event in the existing representation (Zwaan & Radvansky, 1998). This has implications for developmental differences in the comprehension and processing of these sentences: Children are more accurate at comprehending sentences, which describe events in a chronological order compared with sentences that describe events in a reverse order (Clark, 1971).

Previous work has provided two developmental reasons for difficulty with reverse order sentences. First, young children may have a fragile understanding for the meaning of the connective. If so, they will be more likely to use a nonlinguistic strategy to represent the sequence of events based on the assumption that language order maps onto real-world order, rather than using the linguistic information provided by the connective to guide the construction of their mental representation. In line with this, several studies have reported that young children who display a poor knowledge of *before* and *after* comprehend reverse order sentences at below-chance accuracy but are significantly more accurate on chronological sentences (Clark, 1971). The second reason is based on previous adult studies which show that, even when knowledge of temporal connectives is robust, reverse order sentences are still more difficult to process than chronological sentences. This difficulty is attributed to the greater processing costs required to create a chronological mental representation from events that are described in a reverse order relative to when events are described in their actual chronological order (Münte, Schiltz, & Kutas, 1998; Ye et al., 2012). For children, the differences in comprehension of chronological versus reverse order sentences are modulated by the development of memory and vocabulary (Blything, Davies, & Cain, 2015). This set of previous findings motivated the current study to contrast memory capacity-constrained (e.g., Just & Carpenter, 1992) and language-based (e.g., Van Dyke, Johns, & Kukona, 2014) accounts in relation to children’s processing and comprehension of sentences containing temporal connectives.

A memory capacity-constrained framework (e.g., Just & Carpenter, 1992) attributes the difficulties for reverse order sentences to the requirement to hold more information active in working memory, and to the available memory capacity of the individual. Children and adults process complex sentences incrementally, word by word and clause by clause (e.g., Cain & Nash, 2011; Traxler, Bybee, & Pickering, 1997). As a result, reverse order sentences, such as “*Before she drank the juice, she played in the park,*” are more difficult to process than are chronological order sentences, because comprehenders do not process the first occurring event (*played in the park*) until part way through the sentence. As a result, they must then revise their mental representation. Conversely, a chronological order sentence, such as “*She played in the park, before she drank the juice,*” allows incremental construction of the mental representation. Because of the memory demands associated with reverse order sentences, the memory capacity-constrained account would predict that individuals with low memory capacity would experience comprehension difficulties specifically for these constructions. Support for the memory capacity-constrained explanation comes from studies of both adults and children, with the difficulty for reverse order sentences being more pronounced in those who score low on a working memory capacity test (Blything et al., 2015; Münte et al., 1998).

In addition, even when children’s understanding for the connectives is robust enough to no longer rely on a nonlinguistic strategy to understand and represent order (Clark, 1971), the connective might influence processing of these two-clause sentences because it varies the demands on working memory resources. Young children have poorer knowledge of *after* as a connective compared to *before* because it has more complex semantics (Clark, 1971), and is used in ways other than as a connective (e.g., She is only after your money; see Leech, Rayson, & Wilson, 2001). Words that are typically more difficult to learn - as reflected by a late age of acquisition, a low frequency of occurrence, or a high ambiguity in meaning - are processed by adults more slowly and less accurately than their less difficult to learn counterparts (Carroll & White, 1973; Juhasz, 2005). Most important for the predictions of the memory capacity-constrained account, these processing costs are more pronounced in comprehenders with low working memory span compared with comprehenders with high working memory span (Gunter et al., 2003). Therefore, due to the complexity of *after,* sentences containing this connective may be more difficult to process because it is more taxing on working memory resources when activating knowledge of *after* as a temporal connective compared to *before*. Specifically, the influence of the connective on sentence processing should be driven by working memory capacity.

Also, the position of the connective in the sentence may influence the amount of information that must be held active in working memory. By manipulating both order and connective, the position of the connective varies across sentences. For example, *before* occurs in a medial sentence position when events are spoken in a chronological order, but in an initial sentence position when events are spoken in reverse order. The reverse is true for *after* sentences. Position of the connective has also been hypothesized to influence the amount of information held active in working memory. A medial position provides the information of the connective roughly when it is required to link the two meanings of the two adjacent clauses. Conversely, when the connective is provided at the beginning of the sentence, individuals must maintain the meaning of the connective while processing the first clause, and then link the clauses together (Diessel, 2004). In support of the proposal that the connective and its sentence position influence processing, Blything et al. (2015) reported that 4- to 6-year-olds displayed an advantage for chronological order sentences only when the sentence structure did not include these extra features which may increase demands on working memory resources. That finding was modulated by individual memory span, further supporting a memory capacity-constrained account (e.g., Just & Carpenter, 1992).

An alternative hypothesis for how memory influences the processing of these complex sentences is that the effect is actually driven by the quality of language knowledge rather than by the quantity of information that can be maintained within working memory (e.g., Kidd, 2013; Klem et al., 2015; Van Dyke et al., 2014). The language-based account draws on the notion that, rather than being separate systems (Baddeley, 2003; Baddeley & Hitch, 1974), working memory and long-term memory are part of a unitary architecture in which working memory is a temporarily active portion of long term memory (Ericsson & Kintsch, 1995; McElree, 2006). Therefore, the current processing capacity of working memory is determined by the extent to which processing resources are devoted to the retrieval of target concepts from long term memory. That is, the ability to represent information in working memory is modulated by language knowledge. Poor language knowledge is likely to result in a fragile memory representation because the understanding for the meaning of target concepts is less distinct and robust, so the retrieval process is more susceptible to competition from other related concepts. Conversely, rich language knowledge supports the construction of a memory based mental representation because individuals can quickly access and accurately retrieve the precise target concepts. This reduces the likelihood of interference from related concepts, and frees up resources for constructing and maintaining an accurate mental representation.

The language-based account of sentence processing contrasts the memory capacity-constrained account (Just & Carpenter, 1992), which views working memory as independent from language (e.g., Baddeley, 2003; Baddeley & Hitch, 1974). In support of a language-based account of sentence processing, recent research with adults has examined the specificity or distinctness of retrieval cues in the text, for example how well the meaning of the target connective is activated in relation to competing temporal connectives, and how well other words in the sentence are activated in relation to competing words with similar meanings. This work shows that such information, rather than the number of individual text elements that must be held active in memory, can account for why some sentences are more difficult to process than others (Van Dyke et al., 2014; Van Dyke & Johns, 2012; Van Dyke & McElree, 2006).

Research to date has explained children’s difficulty in processing reverse order sentences using the framework of the memory-capacity constrained account (Blything et al., 2015; Pyykkönen & Järvikivi, 2012). However, those studies have used tasks that measure only response accuracy, in which children as young as 6 to 7 years old can perform at ceiling. These findings motivate the need for a more sensitive assessment of children’s sentence processing to study developmental and individual differences in performance. Studies of adults, for whom response accuracy is at ceiling, have used EEG and fMRI to index real-time processing (Münte et al., 1998; Ye et al., 2012). This work demonstrates differences in the effort required to process chronological and reverse order sentences. Such findings have been explained within a memory-based account: reverse order sentences place higher demands on working memory. However, those studies used stimuli in which the connective was presented only in the sentence initial position, such that connective (*before*, *after*) was confounded with event order. This work has not included a design that compares order effects in sentences linked by both *before* and *after*. Further, the only previous studies that have examined online processing of these sentences have not included children, so they do not speak to developmental improvements. A fully factorial design is particularly important in developmental studies because children display developmental differences in their understanding of *before* and *after* (Clark, 1971).

The current study was motivated by our review of previous research on children’s and adult’s processing of multiple clause sentences including temporal connectives, to examine the role of memory and language in children’s comprehension of such sentences. We measured the speed of children’s responses using a touch screen comprehension task (for use of this method with preschool children, see Friend, Schmitt, & Simpson, 2012; Möhring, Newcombe, & Frick, 2014), in addition to response accuracy. Here, we provided strict training and practice instructions to encourage speeded responses. Slower responses can be interpreted as a reflection of processing difficulties, which relate to the extra time needed to construct and revise a mental representation (Cain & Nash, 2011; Just, Carpenter, & Woolley, 1982; Pérez, Paolieri, Macizo, & Bajo, 2014; Zwaan & Radvansky, 1998).

In addition to studying both accuracy and the time taken to make a response, our study differs from previous developmental studies by the nature of the task instructions. Pyykkönen and Järvikivi (2012) asked 8- to 12-year-olds to read a sentence reporting two events and to then indicate which occurred first or whether they occurred at the same time. Even the oldest children were not at ceiling. In a study of the comprehension of similar sentences by much younger children, 6- to 7-year-olds were close to ceiling (Blything et al., 2015). Procedural differences between these studies may explain the age differences in reported competence: Blything et al. (2015) minimized processing demands by using a simple forced-choice touch screen comprehension task in which children were asked to select which event happened first from two images of the actions that were narrated in the sentence. However, Blything et al.’s (2015) “what happened first” instruction may have artificially increased accurate responses for (more complex) reverse order sentences. When children hear a two-clause sentence, the most recently heard event will be more recently activated in the child’s memory than the first mentioned event. If children are asked “what happened first,” the most recent event maps onto the answer for reverse order sentences but not chronological sentences. This could boost response accuracy for reverse order sentences. By asking which event happened last, we can investigate whether children display the same levels and patterns of accuracy as found in previous studies, with a different set of instructions, and in so doing assess the reproducibility of the main findings.

## The Current Study

Children listened to a two-clause sentence containing *before* or *after,* with events narrated either in a chronological or reverse order. During the narration, an animation of the event in each clause was shown, separately, on a touch screen monitor. Children were then asked to touch the picture that represented which of the two events happened last. We did not explicitly manipulate the position of the connective but it varied by the nature of our two within-subject factors: order and connective. Therefore, like others (e.g., Pyykkönen & Järvikivi, 2012), we can also relate our findings to connective position in the sentence.

We first hypothesized that the reason for the youngest children’s poor comprehension would be that they use a nonlinguistic strategy to compensate for a fragile understanding of the connective (Clark, 1971). Evidence for this would come from above chance performance for chronological sentences, but not for reverse order sentences. For the older children, we predicted a different pattern of performance, because they were expected to have more robust knowledge of the specific meaning of the connectives. Specifically, we expected these children to perform above chance for all sentence types, reflecting their ability to accurately encode the connective. However, we predicted that their accuracy for reverse order sentences would be lower than that for chronological order sentences, because of the higher processing demands of this sentence type (Just & Carpenter, 1992; Van Dyke et al., 2014).

Our second and third hypotheses relate to two different accounts: whether memory (Just & Carpenter, 1992) or language knowledge (e.g., Van Dyke et al., 2014) best explains processing difficulties. As memory and language skills both typically improve within the age range of interest, we also predict that whichever skill best explains performance should also explain unique variance over and above the effects of age, thus accounting for developmental improvements. Our use of a timed response measure, in addition to accuracy, provides a sensitive means to assess whether different sentence structures differ in processing ease, as has been found for adults (Münte et al., 1998; Ye et al., 2012).

If a memory capacity-constrained account (e.g., Just & Carpenter, 1992) best explains processing difficulties, children should be more accurate and faster to respond to sentences that place the least demands on working memory. This account predicts the best performance for sentences with a chronological order that are linked by *before* (medial position) because these permit incremental word by word processing. All other sentence combinations (before-reverse, after-chronological, and after-reverse) carry two features that increase the amount of information that must be held in working memory (reverse order, more difficult connective, initial position). Critically, this pattern of performance will be predicted by an independent measure of memory.

If a language-based account (e.g., Van Dyke et al., 2014) best explains processing difficulties, then language knowledge, as measured by performance across connective (*before*, *after*) and by an independent measure of vocabulary, should modulate how well children can process and comprehend sentence structures that require more computational effort. More specifically, we would expect slower and less accurate responses to reverse order sentences linked by *after*, and for the pattern of performance to be driven by our measure of vocabulary knowledge. Critically, the influence of these measures of language knowledge would be expected to override the effects of working memory that would be proposed by the memory capacity account (Just & Carpenter, 1992; as demonstrated by Van Dyke et al., 2014).

Note that the influence of connective knowledge that is proposed by a language-based account of sentence processing (Van Dyke et al., 2014) differs to that proposed by the first (nonlinguistic strategy) hypothesis (Clark, 1971). The first hypothesis focuses on whether young children display below-chance accuracy for reverse order sentences: this would be a result of using a nonlinguistic strategy, which is in turn a result of not having a basic appreciation for the meaning of the connective. Conversely, the language-based account of sentence processing (Van Dyke et al., 2014) relates to when children perform above*-*chance at all sentence structures. Therefore, it focuses on the period that follows children’s appreciation for the meaning of the connective, which is a later period of interest to the first hypothesis and relates to a more fine-grained understanding of the connective that can be used to contrast only the predictions of a memory capacity-constrained account (Just & Carpenter, 1992).

## Method

### Participants

The sample comprised 108 children aged 3 to 7 years from schools in socially mixed catchment areas of North West England. There were 27 3- to 4-year-olds (aged 3;7–4;6, 16 boys), 28 4- to 5-year-olds (aged 4;8–5;7, 15 boys), 27 5- to 6-year-olds (aged 5;8 to 6;6, 15 boys), and 26 6- to 7-year-olds (aged 6;7 to 7;8, 11 boys). Data collection took place between March and June 2015. Written parental consent was obtained for all children, and assent was obtained from all children prior to assessment sessions. All children were native English speakers with no reported language disabilities.

### Materials and Procedure

All children completed assessments of connective comprehension, memory, and receptive vocabulary. The connectives task was administered over two separate sessions. Each session lasted no longer than 15 min. One session included the vocabulary assessment, the other the memory assessment.

#### Connective comprehension task

Comprehension of *before* and *after* was measured using a touch-screen comprehension task. There were 32 sentences that reported events that are arbitrarily related (e.g., He put on the socks, before he ate the burger; see Blything et al., 2015). These 32 two-clause sequences were counterbalanced across four lists so that they each represented one of four sentence constructions that vary by order of mention of events (chronological or reverse) and connective type (*before, after*). The four sentence constructions are shown in Table 1.[Table-anchor tbl1]

We created animated cartoons using Anime Studio Pro 9.1 (Smith Micro Software, 2012). Each cartoon depicted the actor, action and object of the event represented by a clause (e.g., Tom putting on socks; Tom eating a pie). For each item, the animations were presented in a sequential order with the animation on the right hand side of the screen shown first, followed by the animation on the left hand side of the screen. The presentation of the two animations was counterbalanced by both order of appearance and side of presentation. First, the animations were presented to the children. A recorded instruction was then played over headphones (“Listen carefully and touch the thing Tom/Sue did last”), followed by a narration of the sentence itself (e.g., “*Tom/Sue put on the socks before he or she ate the pie*”). A response window was opened with a short beep and was closed by a blank screen once the child had responded.

Practice trial instructions emphasized the importance of making judgments based solely on the meaning of the narrated sentence, not the visual stimuli. These practice trials happened prior to both of the sessions, so that children would be more attentive to the purpose of the task and therefore remember these instructions more easily. One sample *t* tests revealed no significant preference for order or side of presentation (*p*s > .15).

The experiment was run using the PsyScript 3.2.1 (Slavin, 2013) scripting environment on a Macintosh laptop connected to a touch-screen monitor. Items were presented in a random order and no experimental conditions were presented twice on a run at any point, preventing potential priming effects (e.g., Allen, Haywood, Rajendran, & Branigan, 2011). A response was recorded as correct when the child touched the event that was described as happening last. Response time (RT) was the time between the audio beep following the sentence narration and the child’s response.

#### Vocabulary

Our measure of receptive vocabulary was the British Picture Vocabulary Scales—III (Dunn, Dunn, Styles, & Sewell, 2009), in which children have to point to one of four pictures that best illustrates the meaning of a word spoken aloud by the researcher. Testing was discontinued when a specified number of errors had been made, as per the guidelines in the manual. Raw vocabulary scores demonstrated age-related improvements: 3- to 4-year-olds = 64.85 (7.99); 4- to 5-year-olds = 78.71 (7.34); 5- to 6-year-olds = 91.26 (6.74); 6- to 7-year-olds = 98.67 (8.56). All children had a standardized score above 85 and the mean scores (*SD*) indicate that each age group was performing at an age-appropriate level: 3- to 4-year-olds = 108.89 (7.44); 4- to 5-year-olds = 104.43 (8.36); 5- to 6-year-olds = 100.56 (5.62); and 6- to 7-year-olds = 98.38 (7.44).

#### Memory

Each child completed the digit span subtest from the Working Memory Battery for Children (Pickering & Gathercole, 2001) to assess memory. This is the most suitable assessment of memory for our age range, because 4-year-olds perform at floor on more complex measures of working memory (Gathercole, Pickering, Ambridge, & Wearing, 2004). In this task, children were asked to recall a string of digits in the same order that they were spoken by the experimenter. The easiest level comprises strings of two digits, and the number of items in the string is increased once three trials on level were answered correctly. Raw scores were used for the analysis. The raw memory scores (means and standard deviations) demonstrated age-related improvements: 3- to 4-year-olds = 19.11 (3.23); 4- to 5-year-olds = 22.71 (3.14); 5- to 6-year-olds = 25.78 (3.99); 6- to 7-year-olds = 26.81 (3.74). In addition, the standardized scores of memory were within the normal range of 85–115 for each age group: 4- to 5-year-olds = 103.86 (11.00); 5- to 6-year-olds = 108.70 (14.32); and 6- to 7-year-olds = 106.73 (15.84). Standardized scores are not provided for 3- to 4-year-olds. The test–retest reliability reported in the manual for children aged 5 to 7 years is good (*r* = .81).

### Design

A 4 × 2 × 2 mixed design was used. The between-subjects independent variable was age group (3–4, 4–5, 5–6, and 6–7 years) and the within-subjects variables were order (chronological, reverse order) and connective type (*before*, *after*). By manipulating order and connective, we also by nature varied the position of the connective (see Table 1). The dependent variables were accuracy and response times.

## Results

We report the results for accuracy and RTs separately. For each, a series of generalized linear mixed-effects models (GLMMs; Baayen, Davidson, & Bates, 2008) were fitted to the data in the R statistics environment (R Core Team, 2014) using glmer (for the binomial accuracy dependent variable) and lmer (for the continuous RT dependent variable) from package lme4 (Bates, Maechler, & Bolker, 2014). This method is essentially an extension of logistic regression, such that it allows both subject and item effects to be simultaneously treated as random. In other words, a GLMM simultaneously controls for (error) variance that is unexpectedly caused by specific items and specific participants rather than by the fixed effects themselves.

The aim for each model was to have a maximal random effects structure: random intercepts for subjects and items, and random slopes where applicable to the design (Barr, Levy, Scheepers, & Tily, 2013). However, this process highlighted the problems associated with obtaining a maximum model that have been recently outlined by Bates, Kliegl, Vasishth, and Baayen (2015). Specifically, the information in typical data (i.e., the number of observations per subject and per item) is not sufficient to support the complexity of maximum models. As a consequence of this, our most complex models failed to converge. Using the recommendations of Bates et al. (2015), fixed and random effects were incrementally added to a minimal model and were justified by using the likelihood ratio test (Pinheiro & Bates, 2000) for comparing models. In addition, the models were pruned so that nonsignificant factors were removed.

### Accuracy Analysis

We removed 10 children from the analysis: 4 who performed at ceiling across the four sentences (100%), 5 who were identified as outliers in by-age by-sentence box plots, and 1 who was identified as the single outlier in by-age box plots of our independent measure of memory. This did not alter the main findings. Therefore, we report the main effects and interactions of memory, vocabulary, age, order and connective on the accuracy of responses by 98 children.

An initial model (Table A1; see the Appendix) was built that only examined the effects of age, order and connective. This showed no difference between accuracy for *before* and *after* sentences, and no interaction effects between variables (all *p*s > .15). Therefore, following recommendations to allow more complex models to be clearly interpretable and to be better supported by the data (see Bates et al., 2015), these nonsignificant effects were pruned. The pruning of nonsignificant factors did not alter the reported findings (Table A2; see the Appendix) and, together with the removal of data points, ensured a normal distribution of the data that, in turn, allowed convergence of the final reported model that incorporated the effects of memory and vocabulary (see Table 2). Memory and vocabulary were strongly correlated (*r* = .69), so were both centered. The addition of memory, χ^2^(2) = 7.23, *p* < .03, and vocabulary, χ^2^(2) = 7.23, *p* < .03, both improved the fit of the pruned model (Table A2; see the Appendix).[Table-anchor tbl2]

**Figure 1 fig1:**
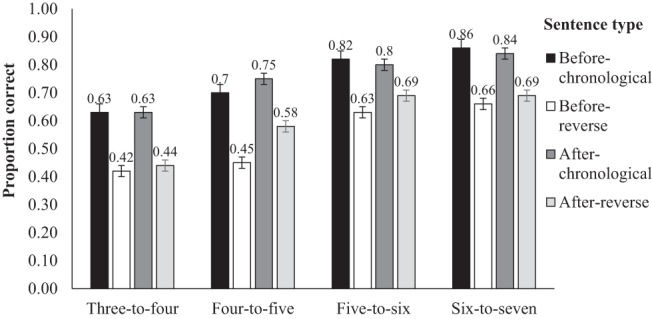
Mean (*SE*) proportion correct for each experimental condition by 3- to 7-year-olds.

The inferential statistics are presented in Table 2. The first column provides the parameter estimates (*b*), which can be interpreted the same way as a regression, such that each shows the change in the log odds accuracy of response associated with each fixed effect on the dependent variable. A positive value indicates that the effect will benefit accuracy, whereas a negative value indicates that the effect will hinder accuracy. The by-age group mean (and standard deviation) accuracy scores for each sentence type are shown in Figure 1. There was a significant and sizable effect of order, because chronological sentences were comprehended more accurately than reverse order sentences. There was also a main effect of memory, because children with higher working memory scores were significantly more accurate on the sentence comprehension task. There were no significant interactions between the variables. The influence of memory was over and above age and vocabulary, which were both nonsignificant. This contrasts with the finding reported in the initial models that had not incorporated memory and vocabulary (Table A1 and Table A2; see the Appendix): These had reported a main effect of age, with each of the three older age groups performing significantly more accurate than the 3- to 4-year-olds. This indicates that the effects of age in those initial models served as a proxy for the role of memory.[Fig-anchor fig1]

We also investigated a possible trade-off between accuracy and RTs. However, the fit of the final reported model (see Table 2), was not improved when RTs were added as a fixed effect covariate, χ^2^(2) = 0.34, *p* < .84 or as item-wise random intercepts, χ^2^(1) = 0.83, *p* < .36. Similarly, these additions did not significantly improve the fit of the models reported in the Appendix (Table A1 and Table A2), all *p*s > .90.

We followed up the main effect of order with one-sample *t* tests to examine whether each age-group performed above chance for chronological compared to reverse order sentences. Our youngest two age groups performed above chance for before-chronological sentences (3- to 4-year olds: *t*[26] = 2.93, *p* < .01; 4- to 5-year olds: *t*[27] = 4.21, *p* < .01) and after-chronological sentences, (3- to 4-year olds: *t*[26] = 2.82, *p* < .01; 4- to 5-year olds: *t*[27] = 5.82, *p* < .01). However, these children were not above chance level for before-reverse sentences (3- to 4-year olds: *t*[26] = −1.60, *p* = .94; 4- to 5-year olds: *t*[27] = −0.85, *p* = .80), or after-reverse sentences (3- to 4-year olds: *t*[26] = −1.17, *p* = .87; 4- to 5-year olds: *t*[27] = −1.38, *p* = .09). This pattern of performance indicates that their inaccuracy for reverse order sentences was likely a result of their fragile understanding for the meaning of *before* and *after.* Conversely, despite performing less accurately for reverse order compared to chronological sentences, our oldest two age-groups still performed above chance for before-reverse sentences (5- to 6-year-olds: *t*[26] = 3.56, *p* < .01; 6- to 7-year-olds: *t*[27] = 3.20, *p* < .01) and after-reverse sentences (5- to 6-year-olds: *t*[26] = 2.88, *p* < .01; 6- to 7-year-olds: *t*[27] = 4.87, *p* < .01). This pattern of results indicates that the older children had a robust appreciation of the meanings of temporal connectives and understood both *before* and *after*. However, their performance was poorer when these connectives were used in sentences that expressed events in reverse order indicating that processing load may be a factor in children’s connective comprehension.

### RT Analysis

**Figure 2 fig2:**
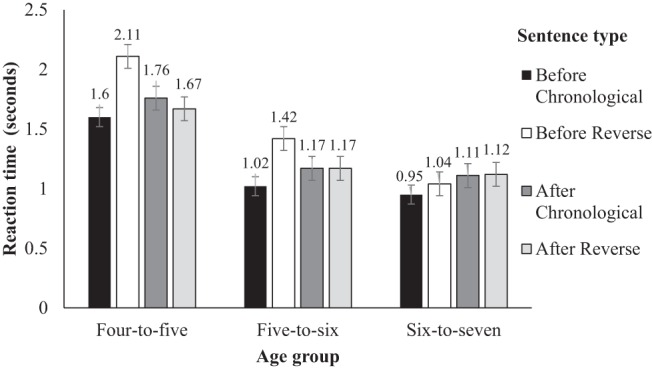
Mean (*SD*) response times (RTs) for each experimental condition by 4- to 7-year-olds.

We did not include responses by 3- to 4-year-olds because their longer RTs suggested that they were not able to follow the instruction to respond as quickly as possible. The 1,816 correct responses by 4- to 7-year-olds were screened following recommendations from Baayen and Milin (2010) to remove potential distortions from the norm and improve the convergence of models. We first removed extreme RTs that exceeded 2.5 standard deviations past the overall mean (49 responses over 9.5 s). Second, we removed remaining outliers that were more than 2.5 standard deviations above the mean response by subject (54 responses) and by item (42 further responses). Thus, a total of 8% of the original data points were removed as outliers. In addition, the data of one 6- to 7-year-old was removed because they were identified as an outlier in by-age box plots of our independent measure of memory. The mean (and standard deviation) RTs in seconds by age-group were 1.75 (1.40) for 4- to-5-year-olds, 1.19 (1.17) for 5- to 6-year-olds, and 1.11 (1.27) for 6- to 7-year-olds. Mean RTs for all correct responses in each experimental condition are presented in Figure 2. Nontransformed means are reported for ease of interpretation. When 3- to- 4-year-olds were screened using this method, their RTs were 2.96 (2.20) s, hence their exclusion.[Fig-anchor fig2]

A square root transformation was used for the inferential analysis so that the data were normally distributed. As in the accuracy analysis, an initial model was built which did not incorporate memory and vocabulary as covariates (Table A4; see the Appendix). However, the RT model was not pruned, because age, order and connective each had either a significant main effect or were involved in an interaction. The same pattern of findings was found in a model of nontransformed RTs (see Table A3; see the Appendix), but our final model (see Table 3) reports the square root transformation because the normal distribution reduced the stress on the model and, in turn, allowed the convergence of the additional effects of (centered) memory and (centered) vocabulary. In GLMMs of data with a continuous dependent variable, it is custom to present t-values and confidence intervals rather than *p* values because, for reasons beyond the current study, the statistical function lmer (from package lme4*;* Bates et al., 2012) does not provide *p* values. Reliably, a significant effect is indicated by a t-value exceeding 2, and when confidence intervals do not pass zero (Baayen, 2008).[Table-anchor tbl3]

Table 3 summarizes the main effects and interactions of memory, age, order and connective on RTs. Similar to the accuracy analysis, there was no main effect of age once memory was added as a covariate, indicating that working memory was driving the developmental improvement in the processing of sentences overall. In contrast to the analysis of the accuracy data, there was a main effect of connective: RTs to sentences with *before* were faster than for sentences with *after*. Also in contrast to the analysis of accuracy data, the main effect of order was not significant: RTs to chronological sentences were not significantly different to those for reverse order sentences.

The main effect of connective was qualified by a three-way interaction between age, order and connective. The influence of age on the effects of order and connective indicates a developmental improvement in the processing of sentences. Therefore, the interaction was broken down by age. This is reported in Table 4 with by age-group models of the effect of order in a subset of each connective. The RTs by 4- to 6-year-old’s were significantly influenced by an interaction between order and connective, whereas older children’s RTs were not. In the 4- to 6-year-olds, there was a main effect of order for *before* sentences, but not for *after* sentences. Specifically, before-chronological sentences were responded to significantly faster than before-reverse sentences, whereas RTs to chronological and reverse order sentences containing *after* did not differ.[Table-anchor tbl4]

In line with the accuracy data, the addition of memory to the model significantly improved the fit of the data, χ2(4) = 11.43, *p* = .02. Children with higher memory capacity made faster (correct) responses overall. Most notably, there was a significant two-way interaction between memory and order, and also one between memory and connective. These interactions indicate that memory predicted the effects of both connective and order. Vocabulary did not improve the fit of the data, χ2(4) = 6.53, *p = .16.* Therefore, we do not report models of RTs that incorporate vocabulary. This indicates that processing times were driven by memory capacity rather than vocabulary per se*.*

## Discussion

This study was designed to identify the reasons why children continue to experience difficulties in comprehending sentences containing *before* and *after* beyond the age that they have begun to display an early competence for these connectives. In general, there were developmental improvements in performance, such that sentences were understood more accurately and processed more quickly by older children. In relation to event order, children were less accurate at comprehending reverse order compared to chronological sentences. Our experimental manipulation of sentence type, together with independent measures of memory and language knowledge, enabled us to test between different theoretical accounts of children’s difficulties with such sentences. The precise pattern of findings indicates different reasons for this effect in younger and older children. As discussed subsequently, the evidence suggests that younger children’s performance with reverse order sentences was limited because they displayed little or no understanding of the connective and instead relied on a nonlinguistic strategy (Clark, 1971). In contrast, older children’s overall performance indicated that they knew the meanings of the two connectives. A consideration of the pattern of performance and how this was related to individual differences in memory and language skills, suggests that older children’s performance was limited by the processing demands of these sentences (Just & Carpenter, 1992; Van Dyke et al., 2014). We first examine the findings of the accuracy analysis and then turn to the analysis of RTs, and discuss why variability in children’s processing of these sentences is best explained by a memory capacity-constrained account (e.g., Just & Carpenter, 1992).

Our findings for response accuracy are convergent with the developmental findings reported by previous studies of children’s comprehension of sentences with temporal connectives (Blything et al., 2015; Clark, 1971; Pyykkönen & Järvikivi, 2012). Children aged 3 to 5 years performed above chance on chronological sentences, but not for reverse order sentences. This difference indicates that they did not take full advantage of the event order that is signaled by the connective and compensated for this by defaulting to an expectation that language order maps onto the actual order of events (Clark, 1971). The 5- to- 7-year-olds performed above chance for all sentence types, which reflects an appreciation for the meaning of the connectives. However, they were in general poorer on reverse order sentences. Because older children displayed an appreciation for the meaning of the connectives, one reason for the lower accuracy for reverse order sentences is that these sentences have higher processing costs (Pyykkönen & Järvikivi, 2012).

Performance on the accuracy task was best explained by memory rather than chronological age or vocabulary. This finding provides partial support for the memory capacity-constrained account (Just & Carpenter, 1992). That is, performance was driven by whether children’s memory capacity was sufficient to cope with the processing demands of our sentences in general. However, the account is only partially supported because the inaccurate comprehension of reverse order compared to chronological sentences did not interact with memory. We argue that the absence of this interaction could be attributed to the task requirement to provide speeded responses. When children are required to respond quickly, they have less time to reflect on and revise the representation that they have constructed and stored in memory (see Marinis, 2010). As a result, the ability to accurately store and manipulate the contents of memory may have a weaker influence on accuracy. Therefore, we turn to our RT measure, to better understand our pattern of data and the processing difficulties experienced by children with these sentence types.

RTs were analyzed for only correct responses to determine if different connectives or structures differed in ease of processing. Thus, the pattern of data cannot be compared directly with the accuracy data. The RT analyses indicate that, even when sentences with temporal connectives are comprehended correctly, some are more difficult to process than others (e.g., Cain & Nash, 2011; Ye et al., 2012). The RT data support the memory capacity-constrained account (Just & Carpenter, 1992). Children responded most quickly to chronological order sentences linked by *before* (medial position), which allow incremental word by word processing; and more slowly to before-reverse sentences, which do not afford incremental processing. There was no effect of order for sentences containing *after.* After-chronological sentences (initial position, later acquired connective) sentences and after-reverse sentences (reverse order, later acquired connective) each carry two features associated with taxing information to be held in working memory, and do not permit incremental processing. This may be the reason for the absence of RT differences between these two sentence types.

Importantly, the incorporation of memory significantly improved the fit of the model for RTs, whereas vocabulary did not. Moreover, the main effect of age was no longer significant when memory was added to the model. Instead, the main effect of memory can account for developmental improvements in the processing of these sentences. This suggests that, as in the accuracy findings, age effects were partly a proxy for the influence of memory. Of particular note, the variation in RTs across our sentence structures was predicted by our independent measure of memory span. This indicates that demands on working memory are driving these effects. That is, children with higher working memory spans are better able to cope with the higher memory demands of difficult sentences, and so experience fewer problems, as do adults (Just & Carpenter, 1992).

In turn, the support we provide for a memory capacity-constrained account of sentence processing informs and maps onto our understanding of how the temporal information in these sentences is mentally represented (Gennari, 2004; Zwaan & Radvansky, 1998). We interpret the slower responses to sentences that do not afford incremental processing as a reflection of processing difficulties that relate to the extra time needed to construct and revise a mental representation (Cain & Nash, 2011; Just et al., 1982; Pérez et al., 2014; Zwaan & Radvansky, 1998). Those sentences carry additional memory processing demands because more information must be maintained in working memory while the mental representation is revised. It follows that children who have lower working memory capacity will be less capable of revising the mental representation into the desired accurate linear order. This provides additional support to previous studies that have attributed children’s inaccuracy with these sentence structures to a difficulty in mentally representing sentences that carry higher memory processing demands (Blything et al., 2015; Pyykkönen & Järvikivi, 2012).

Of course, we should not dismiss language effects per se. For example, the advantage for chronological sentences displayed by the younger children is a result of their below-chance accuracy for reverse order sentences. This suggests that when children do not have an appreciation for the meaning of a temporal connective, they will use a nonlinguistic strategy to understand and represent the relation between two events (Clark, 1971). However, these findings are not relevant to the language-based account of processing (Van Dyke et al., 2014), which focuses on a more fine-grained understanding of the connective (and the other words in the sentence) in the immediate years that follow an appreciation for its meaning. We did report an effect of language knowledge on processing: *before* sentences had faster RTs than *after* sentences. However, children with a higher working memory capacity were less likely to display such effects. Therefore, these connective effects are interpreted in line with a memory capacity-constrained framework (Just & Carpenter, 1992), such that sentences linked by the more complex connective *after* carry additional demands on working memory compared to sentences linked by *before* (Clark, 1971; Leech et al., 2001). This fits the prediction that chronological sentences linked by *before* are processed most easily because it is the only sentence structure that does not carry any additional features that increase the amount of information to be held in working memory (easier connective, chronological order, medial position).

A strength of our design was the manipulation of both memory and language processing requirements of our stimuli, in addition to the use of independent measures of memory and language to relate to performance. It is worth noting that language research is becoming increasingly aware of the need use an intensive battery of measures for individual differences in skills such as memory and vocabulary (Language and Reading Research Consortium, 2015). We selected a single measure of short-term memory (STM) with a low semantic load to better disentangle the effects of memory and language, noting that memory measures with greater semantic content are more strongly related to language processing ability in young children than digit based tasks (Cain, 2006; Seigneuric, Ehrlich, Oakhill, & Yuill, 2000). Because of our age range, we were not able to use a measure of complex memory span (Gathercole et al., 2004) and note that such a measure may be more strongly related to language processing than our STM measure (Daneman & Merikle, 1996). Similarly, we measured only the breadth of vocabulary (i.e., number of words known or not known), a measure used frequently with our age cohort (e.g., Silva & Cain, 2015). However, depth of vocabulary knowledge (i.e., the richness of knowledge for a particular word) is also highly predictive of comprehension ability (Cain & Oakhill, 2014; Ouellette, 2006). Therefore, future work should explore the sensitivity and inclusion of more complex measures of memory and vocabulary when assessing the relation between these skills and language processing to provide a more accurate assessment of these constructs to relate to sentence comprehension.

It is also worth noting that the accuracy findings inform us of the importance of the nature of the task itself. Children were less accurate overall relative to previous studies of the same age group (e.g., Blything et al., 2015). This is most likely a result of the requirement for children to produce speeded responses. However, relative to previous studies, children also displayed lower accuracy for reverse order sentences. That poor performance cannot be attributed to the speeded instructions alone, because accuracy for chronological sentences was equivalent to previous studies.^1^ In line with our predictions, we attribute this difference to the use of the “what happened last” question. Therefore, the current study suggests that, in forced-choice paradigms for these sentences, accuracy may be distorted by false positive answers whereby children are more likely to choose the target answer because it maps onto the event that had been most recently activated in memory. This highlights the motivation of the current study to inform existing accuracy data with a measure of processing ease (RTs) in addition to accuracy.

This is the first study to report a measure that indicates how efficiently children process two-clause sentences containing *before* and *after*. That is, it takes the first step to supporting previous forced-choice accuracy studies that have attributed children’s inaccurate comprehension to a difficulty in representing sentences that do not afford incremental processing (Blything et al., 2015; Pyykkönen & Järvikivi, 2012). The specific measure was chosen because the paradigm was analogous to the touch screen comprehension task used by Blything et al. (2015), The average RTs were well within the range of those that have been previously reported by other touch screen paradigms as a reflection of children’s mental representations (Möhring et al., 2014); and previous studies have also interpreted RTs to comprehension accuracy tasks as a reflection of the time needed to construct and revise a mental representation (e.g., Cain & Nash, 2011; Pérez et al., 2014; Zwaan & Radvansky, 1998). However, in order to gain a full picture of how children process these sentences, further research must assess real time moment by moment processing in sentence comprehension (and production). For example, the reason that our memory measures were less likely to influence RTs in children with increasing age, may be that, at their more advanced developmental stage, they are more capable of revising the mental representation during sentence presentation. A paradigm that included measurement of ERPs might usefully indicate where the cognitive demands were greatest and whether processing effort for particular sentence regions are more strongly related to independent measures of memory, as has been shown with adults (Münte et al., 1998).

Overall, our analyses demonstrate age-related differences in 3- to 7-year-olds’ understanding of temporal connectives (e.g., Clark, 1971). The pattern of findings supports the conclusion that the 3- to 5-year olds were inaccurate because they had a poor appreciation for the meaning of the connectives and so could not appropriately use the linguistic information about temporal order. The 5- to 7-year-olds demonstrated a robust understanding of the connective but displayed evidence of processing difficulties. Our critical processing time measure provided evidence that the processing difficulty can be attributed to the memory load of the sentence structure and to the available memory resources of the individual (Just & Carpenter, 1992). Finally, we emphasize the need for future studies to test the generalization of this conclusion with different independent measures of memory, more comprehension assessments of vocabulary knowledge, and online paradigms that provide an indicator of processing efficiency during the comprehension of the sentence itself.

## Figures and Tables

**Table 1 tbl1:** Sentence Conditions

Before		After	
Chronological	Reverse	Chronological	Reverse
He put on the socks, before he ate the pie.	Before he ate the pie, he put on the socks.	After he put on the socks, he ate the pie.	He ate the pie, after he put on the socks.

**Table 2 tbl2:** Main Effect and Interactions of Memory, Vocabulary, Age, and Order on the Proportion of Correct Answers by 3- to 7-Year-Olds

				CI		
Main model	*M* (*b*)	*SE*	*z*	2.5%	97.5%	*p*(>|*z*)
Fixed effects						
(Intercept)	.20	.22	.90	−.24	.64	.37
Memory	**.06**	**.03**	**2.11**	**<.01**	**.11**	**.04**
Vocabulary	.02	.01	1.56	<.01	.04	.12
Four-to-five	.02	.23	.09	−.44	.48	.93
Five-to-six	.14	.34	.40	−.53	.80	.69
Six-to-seven	.28	.38	.74	−.46	1.02	.46
Order	**.91**	**.10**	**9.12**	**.71**	**1.10**	**<.01**
Memory: Order	−.03	.03	−.92	−.09	.03	.36
Vocabulary: Order	.01	.01	.57	−.01	.02	.57
					Variance	*SD*
Random effects						
Participant (intercept)					.53	.72
Participant.1 (slope) order					.29	.54
*Note.* Number of observations = 3,136; groups = 98 participants. Values in bold indicate that the predictor is significant at *p* < .05 or better.						

**Table 3 tbl3:** Main Effect and Interactions of Memory, Age, Order, and Connective on Response Times (With Square Root Transformation) to Correct Answers by 4- to 7-Year-Olds

				CI	
Main model	*M* (*b*)	*SE*	*t*	2.5%	97.5%
Fixed effects					
(Intercept)	1.10	.07	16.18	.97	1.24
Memory	**−.03**	**.01**	**−3.06**	**−.05**	**−.01**
Five-to-six	−.06	.09	−.59	−.24	.13
Six-to-seven	−.13	.10	−1.37	−.32	.06
Order	.09	.06	1.43	−.03	.21
Connective	**.28**	**.07**	**3.86**	**.14**	**.42**
Memory: Order	**.02**	**.01**	**2.03**	**.00**	**.04**
Five-to-six: Order	−.15	.09	−1.75	−.32	.02
Six-to-seven: Order	−.13	.09	−1.56	−.30	.03
Memory: Connective	**.02**	**.01**	**2.49**	**.01**	**.04**
Five-to-six: Connective	**−.25**	**.09**	**−2.62**	**−.43**	**−.06**
Six-to-seven: Connective	**−.33**	**.10**	**−3.38**	**−.52**	**−.14**
Order: Connective	**−.32**	**.09**	**−3.49**	**−.50**	**−.14**
Memory: Order; connective	−.02	.01	−1.76	−.05	<.01
Five-to-six: Order; connective	.22	.12	1.75	−.03	.46
Six-to-seven: Order; connective	**.33**	**.13**	**2.60**	**.08**	**.58**
				Variance	*SD*
Random effects					
Participant (intercept)				.06	.24
Item (intercept)				<.01	.07
Residual				.21	.46
*Note.* Number of observations = 1,648; groups = 80 participants and 64 items. Values in bold indicate that the predictor is significant at *p* < .05 or better.					

**Table 4 tbl4:** Simple Effects Age-Group Models of the Effect of Order by Connective Type on Response Times (With Square Root Transformation) to Correct Answers

	Age 4–5					Age 5–6					Age 6–7				
				CI					CI					CI	
Fixed and Random effects	(*b*)	*SE*	t	2.5%	97.5%	(*b*)	*SE*	*t*	2.5%	97.5%	(*b*)	*SE*	t	2.5%	97.5%
	Before														
Fixed effects															
(Intercept)	1.38	.06	22.42	1.26	1.50	1.08	.06	16.97	.95	1.20	.90	.06	14.44	.78	1.02
Order	**−.22**	**.06**	**−3.35**	**−.34**	**−.09**	**−.18**	**.06**	**−3.21**	**−.28**	**−.07**	−.04	.05	−.67	−.14	.07
				Variance	*SD*				Variance	*SD*				Variance	*SD*
Random effects															
Participant				.02	.18				.06	.24				.01	.01
Item				.03	.12				.00	.06				.01	.01
Residual				.2	.45				.21	.46				.01	.01
	After														
Fixed effects															
(Intercept)	1.19	.06	19.72	1.07	1.30	1.02	.06	16.10	.90	1.15	.94	.08	12.34	.79	1.09
Order	.04	.06	.61	−.08	.16	−.04	.05	−.72	−.14	.06	−.02	.05	−.43	−.13	.08
				Variance	*SD*				Variance	*SD*				Variance	*SD*
Random effects														
Participant				.04	.19				.06	.25				.02	.12
Item				<.01	<.01				<.01	.06				.10	.32
Residual				.25	.5				.18	.42				.19	.44
*Note.* Number of observations for ages 4 to 5 *before* models = 230; groups = 28 participants and 64 items. Number of observations for ages 4 to 5 *after* models = 267; groups = 28 participants and 63 items. Number of observations for ages 5 to 6 *before* models = 292; groups = 27 participants and 64 items. Number of observations for ages 5 to 6 *after* models = 267; groups = 27 participants and 64 items. Number of observations for ages 6 to 7 *before* models = 282; groups = 25 participants and 63 items. Number of observations for ages 6 to 7 *after* models = 290; groups = 25 participants and 64 items. Values in bold indicate that the predictor is significant at *p* < .05 or better.															

**Table A1 tbl5:** Justification for Pruning the Nonsignificant Main Effect and Interactions of Age, Order, and Connective on the Proportion of Correct Answers by 3- to 7-Year-Olds

				CI		
Main model	*M* (*b*)	*SE*	*z*	2.5%	97.5%	*p*(>|*z*)
Fixed effects						
(Intercept)	−.25	.22	−1.14	−.68	.18	.25
Four-to-five	**.64**	**.31**	**2.04**	**.02**	**1.25**	**.04**
Five-to-six	**1.21**	**.33**	**3.67**	**.56**	**1.85**	**<.01**
Six-to-seven	**1.21**	**.35**	**3.51**	**.54**	**1.89**	**<.01**
Order	**.81**	**.23**	**3.49**	**.35**	**1.26**	**<.01**
Connective	−.13	.25	−.51	−.61	.36	.61
Four-to-five: Order	.01	.34	.04	−.65	.67	.97
Five-to-six: Order	−.27	.36	−.74	−.97	.44	.46
Six-to-seven: Order	.02	.39	.06	−.74	.79	.95
Four-to-five: Connective	−.51	.35	−1.45	−1.21	.18	.15
Five-to-six: Connective	−.23	.37	−.63	−.95	.49	.53
Six-to-seven: Connective	−.03	.39	−.07	−.79	.74	.95
Order: Connective	.14	.29	.49	−.42	.71	.62
Four-to five: Order; connective	.29	.42	.68	−.54	1.12	.49
Five-to-six: Order; connective	.39	.45	.86	−.50	1.28	.39
Six-to-seven: Order; connective	.25	.50	.49	−.74	1.24	.62
					Variance	*SD*
Random effects						
Participant (intercept)					.80	.90
Participant (Slope 1): Order					.33	.57
Participant (Slope 2): Connective					.54	.73
*Note.* Number of observations = 3,136; groups = 98 participants. Values in bold indicate that the predictor is significant at *p* < .05 or better.						

**Table A2 tbl6:** Main Effect of Age and Order on the Proportion of Correct Answers by 3- to 7-Year-Olds

				CI		
Main model	*M* (*b*)	*SE*	*z*	2.5%	97.5%	*p*(>|*z*)
Fixed effects						
(Intercept)	−.34	.15	−2.22	−.64	−.04	.03
Four-to-five	**.47**	**.20**	**2.37**	**.08**	**.86**	**.02**
Five-to-six	**1.02**	**.21**	**4.91**	**.62**	**1.43**	**<.01**
Six-to-seven	**1.25**	**.22**	**5.60**	**.81**	**1.69**	**<.01**
Order	**.90**	**.10**	**9.12**	**.71**	**1.10**	**<.01**
					Variance	*SD*
Random effects						
Participant (intercept)					.62	.79
Participant.1 (slope) order					.30	.55
*Note.* Number of observations = 3,136; groups = 98 participants. Values in bold indicate that the predictor is significant at *p* < .05 or better.						

**Table A3 tbl7:** Main Effect and Interactions of Age, Order, and Connective on Response Times (Without Square Root Transformation) to Correct Answers by 4- to 7-Year-Olds

				CI	
Main model	*M* (*b*)	*SE*	*t*	2.5%	97.5%
Fixed effects					
(Intercept)	1.66	.17	9.62	1.32	2.00
Five-to-six	−.35	.24	−1.47	−.82	.12
Six-to-seven	−.46	.24	−1.91	−.93	.01
Order	.12	.16	.75	−.19	.43
Connective	**.56**	**.17**	**3.33**	**.23**	**.88**
Five-to-six: Order	−.22	.22	−1.01	−.65	.21
Six-to-seven: Order	−.19	.22	−.86	−.62	.24
Five-to-six: Connective	−.41	.22	−1.89	−.84	.01
Six-to-seven: Connective	**−.60**	**.22**	**−2.72**	**−1.03**	**−.17**
Order: Connective	**−.69**	**.21**	**−3.27**	**−1.10**	**−.28**
Five-to-Six: Order; connective	.44	.28	1.56	−.11	1.00
Six-to-Seven: Order; connective	**.60**	**.28**	**2.10**	**.04**	**1.15**
				Variance	*SD*
Random effects					
Participant (intercept)				.47	.69
Participant (Slope 1): Order				.12	.35
Participant (Slope 2): Connective				.03	.18
Item (intercept)				.03	.18
Residual				1.24	1.11
*Note.* Number of observations = 1,648; groups = 80 participants and 64 items. Values in bold indicate that the predictor is significant at *p* < .05 or better.					

**Table A4 tbl8:** Main Effect and Interactions of Age, Order, and Connective on Response Times (With Square Root Transformation) to Correct Answers by 4- to 7-Year-Olds

Main model	*M* (*b*)	*SE*	*t*	CI	
				2.5%	97.5%
Fixed effects					
(Intercept)	1.18	.06	18.52	1.05	1.30
Five-to-six	−.16	.09	−1.83	−.33	.01
Six-to-seven	**−.24**	**.09**	**−2.75**	**−.42**	**−.07**
Order	.05	.06	.78	−.07	.16
Connective	**.22**	**.07**	**3.20**	**.08**	**.35**
Five-to-six: Order	−.08	.08	−1.04	−.24	.07
Six-to-seven: Order	−.06	.08	−.81	−.22	.09
Five-to-six: Connective	−.16	.09	−1.82	−.33	.01
Six-to-seven: Connective	**−.23**	**.09**	**−2.62**	**−.41**	**−.06**
Order: Connective	**−.26**	**.09**	**−3.08**	**−.43**	**−.10**
Five-to-six: Order; connective	.14	.12	1.17	−.09	.36
Six-to-seven: Order; connective	**.24**	**.12**	**2.05**	**.01**	**.46**
				Variance	*SD*
Random effects					
Participant (intercept)				.06	.24
Item (intercept)				<.01	.07
Residual				.21	.46
*Note.* Number of observations = 1,648; groups = 80 participants and 64 items. Values in bold indicate that the predictor is significant at *p* < .05 or better.					

## Footnotes

1 Older children did display a slight reduction in accuracy for before-chronological sentences relative to the ceiling level achieved in a recent study by Blything et al. (2015), but these reductions are likely a result of ceiling performance being a less realistic opportunity with speeded responses.

## Appendix

[Table-anchor tbl5][Table-anchor tbl6][Table-anchor tbl7][Table-anchor tbl8]
